# Chemical Fingerprinting of Seeds of Some *Salvia* Species in Turkey by Using GC-MS and FTIR

**DOI:** 10.3390/foods8040118

**Published:** 2019-04-04

**Authors:** Eray Tulukcu, Nur Cebi, Osman Sagdic

**Affiliations:** 1 Technical Vocational School of Higher Education, Program of Medical Aromatic Plants, Selcuk University, 42500 Cumra, Konya, Turkey; eraytulukcu@selcuk.edu.tr; 2 Food Engineering Department, Chemical and Metallurgical Engineering Faculty, Yıldız Technical University, 34210 Istanbul, Turkey; sagdic@gmail.com

**Keywords:** Salvia seed, GC-MS, FTIR, volatile content, chemometrics, HCA, PCA

## Abstract

Six species of *Salvia* seeds cultivated and grown in Cumra/Konya (Turkey) were evaluated using headspace gas chromatography mass spectroscopy (GC-MS) and Fourier transform infrared spectroscopy-attenuated total reflectance (FTIR-ATR) combined chemometrics of hierarchical cluster analysis (HCA) and principal component analysis (PCA). The major volatile compounds in the *Salvia* species are determined as *n*-hexanal (present in seven samples), sabinene (present in three samples), α-pinene (present in 13 samples), α-thujone (present in four samples), borneol (present in 11 samples), linalyl acetate (present in 10 samples), β-pinene (present in 13 samples), camphene (present in 13 samples), α-thujene (present in four samples), 2,4(10)-thujadien (present in two samples), β-myrcene (present in seven samples), limonen (present in 12 samples), 1,8-cineole (eucalyptol) (present in 13 samples) and camphor (present in nine samples). The most abundant (%) volatile compounds among all were detected as α-pinene, camphene, β-pinene and eucalyptol. For the first time, chemometrics of HCA and PCA is applied to FTIR and GC-MS data. The classification of all samples is performed on the basis of their chemical similarities and differences.

## 1. Introduction

*Salvia* has been known as the largest genus of the *Lamiaceae* family. This large genus comprises a wide variety of species through the whole flora. Previous reports showed that approximately 900 species have been known as *Salvia* all across the world. Eighty-six species of *Salvia* (*Lamiaceae*) have been known in Turkey [1] and many species are medicinal and aromatic plants. *Salvia* species (*Lamiaceae*) could be defined to be quite endemic with a high endemism percentage of 45% in the flora of Turkey [2]. The origin of the word *Salvia* dates back to the Latin word “*salvare*” which means “to heal” [3]. Previous scientific reports presented that *Salvia* has health-healing properties such as antiseptic, antipyretic, analgesic, antimicrobial, antioxidant, anticancer, anticholinesterase and anti-inflammatory characteristics, and it is used for herbal teas [3]. Different parts of the *Salvia* plant such as leaves, flowers, roots and seeds may be used for their health benefits. The seeds of *S. hispanica*, *S. sclarea*, *S. umbratica* and *S. viridis* are used for medicinal purposes [3]. Results from previous studies demonstrated a strong and consistent relationship between the genus *Salvia* and antimicrobial, antiviral and cytotoxic effects [4]. In Turkey, *Salvia* species are mainly consumed in the form of herbal teas with the aim of curing the common cold, and *Salvia* has applications in treating some diseases which cause pain in different parts of the body such as the stomach and liver [5]. *Salvia* seeds have been known to have an important nutritional composition. Seeds contain fat, protein, fibers, minerals and phenolic compounds. Thus, they are indicated as sources of fats, proteins, antioxidants and dietary fibers [6]. For instance, *Salvia hispanica* L. has 17–20% protein content and 25–38% fat content with approximately 60% linoleic acid content [7]. Additionally, previous studies reported that it is possible to isolate important new and previously known compounds such as abietane, clerodane, pimarane diterpenoids, sesterterpenoids, triterpenoids and flavonoids. As clearly seen, *Salvia* seeds may qualify for development of new functional foods, innovative food products and dietary supplements [7]. Along with this growth in the food applications of *Salvia,* there is increasing interest in the economic and nutritional value of *Salvia* species. In recent years, there has been an increasing interest in fingerprinting (characterization of) *Salvia* species [8]. Previous studies showed the chemical properties and compositional and physical characteristics of this valuable seed. However, very few studies have focused on the evaluation of the seeds of *Salvia* species. For instance, Porras-Loaiza et al. (2014) performed a study with the aim of comparing the physical and chemical properties of chia (*Salvia hispanica*) seeds from different Mexican regions [7]. In another study, the main objective was to chemically and nutritionally characterize commercial chia seeds [9]. Similarly, Capitani et al. (2012) characterized the physicochemical and functional properties of meals and fibrous fractions of chia seeds [10]. In another study, Yilmaz et al. (2016) evaluated the rheological characteristics of *Salvia sclarea* seed gum [11]. It is now well established by a variety of studies that *Salvia* seeds have high saturated, monounsaturated and polyunsaturated fatty acids in their composition [3]. It was reported that fingerprinting herbs may be accomplished using chromatographic techniques. Findings suggested that the application of chromatographic techniques for characterization and identification of herbs has gained recognition from foremost organizations such as the World Health Organization and Food and Drug Administration [8]. Gas chromatography is effectively and preferably used in various sections of the food industry such as process control and quality control with the aim of tracking the composition of volatile compounds [12]. The gas chromatography mass spectroscopy (GC-MS) technique especially could be regarded as the gold standard since the combination of gas chromatography with mass spectroscopy provides opportunities for the identification of unknown components with high accuracy [13]. Another popular and strong fingerprinting technique is known as Fourier transform infrared spectroscopy (FTIR spectroscopy). FTIR spectroscopy is non-destructive and can rapidly obtain biochemical fingerprints that provide reliable information on molecular structure and composition [14]. Because of these advantages, FTIR spectroscopy has been used effectively and successfully with the aim of identification and evaluation of a wide variety of food products. Nowadays, many laboratories have the capability to produce comprehensive data sets by using this instrumental analysis equipment. Chemometrics in statistics is used to evaluate the outcomes obtained by laboratory equipment [15]. Chemometrics may be used effectively and efficiently to determine the hidden relationships between variables, thus providing an opportunity for evaluation of the samples in compliance with their similarities or differences. The most popular and widely used techniques are known as hierarchical cluster analysis (HCA), principal component analysis (PCA) and partial least squares (PLS) [15].

The primary aim of this paper was to evaluate the chemical fingerprints of different *Salvia* species by using GC-MS and FTIR. For the first time in this study, the major volatile compounds of *Salvia* seed species in Konya (Turkey) were identified by using a library of GC-MS. Additionally, HCA and PCA chemometric analyses of the GC-MS and FTIR data were performed for classification of *Salvia* seed species.

## 2. Materials and Methods

### 2.1. Equipment

GC-MS analyses were performed by using a GCMS-QP2010 gas chromatography mass spectrometer system (Shimadzu, Milan, Italy). Commercial libraries were used for identification of detected compounds. Instrument control and data collection were provided using the GCMS Solution software (GCMS Real Time Analysis and GCMS Postrun Analysis). FTIR analyses were performed by using a Bruker Tensor 27 spectrometer (Bremen, Germany) with a KBr beam splitter and a deuterated l-alanine doped triglycene sulphate (DLaTGS) detector. A diamond single-bounce attenuated total reflectance (ATR) accessory was used in all measurements. Instrument control and data acquisition were accomplished by using OPUS version 7.2 for Windows from Bruker Gmbh (Ettlingen, Germany). The retention indices were determined by analyzing an *n*-alkane hydrocarbon mixture (C8–C40 series, Sigma-Aldrich, St. Louis, MO, USA).

### 2.2. Sampling

In our study, six *Salvia* species which were grown in Konya (Turkey) were evaluated using GC-MS and FTIR techniques. These species were *S. triloba* (I and II), *S. officinalis* (I and II), *S. nemorosa* (I), *S. sclarea* (I and II), *S. virgata* (I, II, III and IV) and *S. microstegia* (I and II). The numbers in the brackets represent the same *Salvia* species which were farmed in the different sections of farming field. In total, 13 samples were evaluated in the scope of this research study. Each species was grown and identified by a botany specialist.

### 2.3. Static HS-GC/MS Analysis

Samples were grinded using a spice grinder (Sinbo SCM 2934, Turkey). Then, 3 g of ground sample was transferred into 20 mL headspace vials. Sample loaded vials were heated and agitated for 15 min at 70 °C by the auto sampler system. Headspace autosampler parameters were adjusted to an incubation temperature of 70 °C, incubation time of 15 min; syringe temperature of 70 °C, agitation speed of 500 rpm; injection volume of 500 µL; fill speed of 200 µL/s, pull up delay of 500 ms; injection speed of 350 µL/s; pre-injection delay of 500 ms; and post injection delay of 1500 ms. As mentioned, 0.5 mL of headspace (HS) sample was introduced into the GC-MS system. HS/GC-MS analyses were performed by using a GCMS-QP2010 (Shimadzu, Milan, Italy) combined with a CTC-Combi-PAL-autosampler (Bender and Hobein, Zurich, Switzerland). In the GC-MS analyses, A Restec (Bellefonte, USA) Rtx-5MS fused silica capillary column (30 m × 0.25 mm (internal diameter) 0.25 μm) was used for chromatographic separation. The carrier gas used was helium. Volatile analyses of *Salvia* seeds were performed with some modifications in the method of Rzepa et al. [8]. Gradient analysis was performed using the following temperature program: 40 °C (3 min); 40–176 °C (8 °C/min); and 176 °C (20 min). The temperature of the injector was kept constant at 150 °C. The pressure value and linear velocity were 95.8 kPa and 47.1 cm/s, respectively. The carrier gas flow rate was 1.71 mL/min. The GC–MS interface temperature and ion source temperature were 280 °C and 230 °C, respectively. The mass spectrometer was operated in the selected ion-monitoring mode with an electron impact ionization voltage of 70 eV and data were collected over a range of m/z 35–550. Analyses were performed in duplicate for each sample. The major volatile compounds of *Salvia* species were identified by using a library of GC-MS. Identification is provided by comparison of the mass spectra of the detected volatile compounds with the commercial mass spectra libraries (NIST27 and WILEY7). Quantification was performed on the basis of relative area of the total ion chromatogram (TIC) peaks of volatile compounds. Obtained data were used with the aim of identification of *Salvia* species.

### 2.4. FTIR Spectroscopy Measurements

Grinded *Salvia* seed samples were directly placed upon the diamond ATR crystal and pressed by the ATR accessory. Attenuated total reflectance spectra of all *Salvia* seed species were scanned with a resolution of 4 cm^−1^ and 16 scans were accumulated per spectra. All spectra were obtained in triplicate and the average spectrum of three measurements was calculated for each species. An air background spectrum was obtained prior to each ATR measurement. Spectral acquisitions were gathered by compressing the sample by using the ATR accessory. Ethyl alcohol and warm water were used in the cleaning of ATR cell.

### 2.5. Chemometric Analysis

PCA and HCA analyses were performed for all obtained data (GC-MS and FTIR). For chemometrics, HCA and PCA were performed using the software OPUS Version 7.2 (Bruker, Germany) for FTIR data of *Salvia* species. Similarly, HCA and PCA multivariate analyses were performed by SIMCA 15 (Umetrics, Umea, Sweden) for GC-MS data of *Salvia* species.

## 3. Results and Discussion

### 3.1. Static HS-GC/MS Analysis

**Figure 1:**
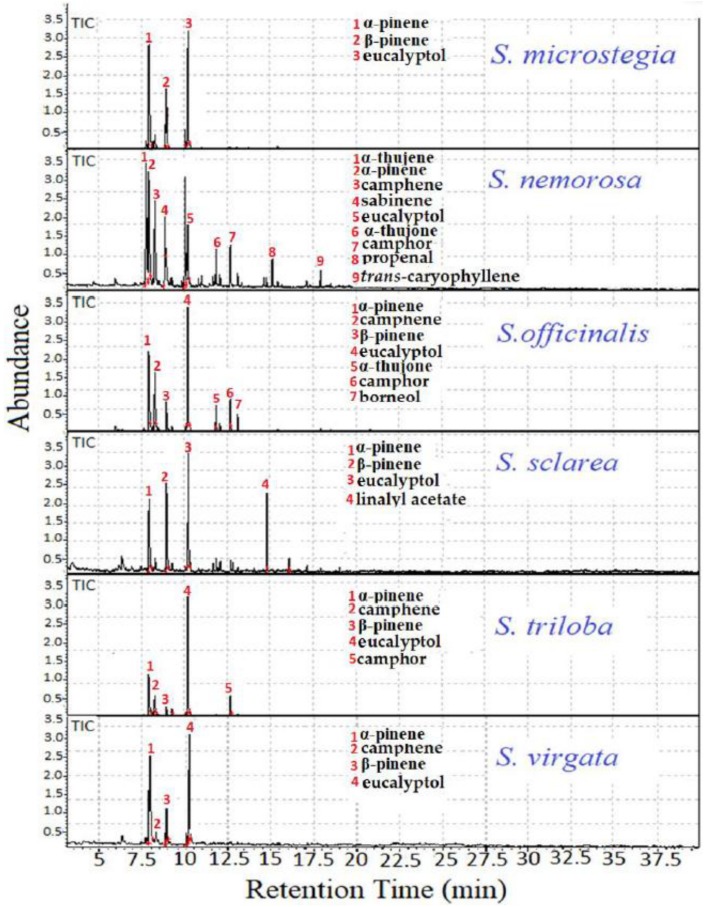
Characteristic gas chromatography mass spectroscopy (GC-MS) total ion chromatogram (TIC) of the seed samples of *Salvia* species (*S. microstegia*, *S. nemorosa*, *S.officinalis*, *S. sclarea*, *S. triloba*, *S. virgata*).

The percentage compositions (area %) of the identified volatile compounds in *Salvia* seeds are presented in Table 1. In total, twenty-eight compounds were identified by comparing the mass spectra of the *Salvia* species to the library of GC-MS. The characteristic total ion chromatograms of different *Salvia* species are presented in Figure 1. The major peaks were numbered, and the names of these peaks are annotated in Figure 1. All of these compounds and their percentage compositions are presented in Table 1. The major volatile compounds in the *Salvia* species are determined as *n*-hexanal (present in seven samples), sabinene (present in three samples), α-pinene (present in 13 samples), α-thujone (present in four samples), borneol (present in 11 samples), linalyl acetate (present in 10 samples), β-pinene (present in 13 samples), camphene (present in 13 samples), α-thujene (present in four samples), 2,4(10)-thujadien (present in two samples), β-myrcene (present in seven samples), limonen (present in 12 samples), 1,8-cineole (eucalyptol) (present in 13 samples) and camphor (present in nine samples). The most abundant (%) volatile compounds among all were detected as α-pinene, camphene, β-pinene and eucalyptol. All of the detected volatile compounds are presented in Table 1. Important studies were carried out for determination of the chemical composition of common sage essential oils. In particular, the bioactive properties of *Salvia* species were evaluated in some of the studies [16,17]. In an important study, camphor, thujone, 1,8 cineole, borneol, -pinen and thujone were determined as the major constituents of sage essential oil [18]. Quite similar results to those found previously were obtained in our study as seen in Table 1. A previous study determined the fingerprints of selected *Salvia* species by using the HS-GC/MS technique, and according to their findings, the major volatile compounds were α,β pinene, thujol and camphor β. Additionally, they found β-myrcene and β-phellandrene in some species and caryophyllene in *S. officinalis* [8]. Our results were quite compatible with the findings of Rzepa et al. (2009); in particular, *trans*-caryophyllene was observed in both samples of the *S. officinalis* species. In another study, 1,8-cineole, borneol, camphor and thujone were listed as the known constituents of *Salvia* species [19]. Distinctive volatile profiles were observed between *Salvia* species in that they contained volatile compounds in different percentages. In our study, while the highest amounts of α-pinene were observed in *S. microstegia,* the lowest amounts were observed in *S. nemorosa*. The highest amount for β-pinene was observed in *S. sclarea*. The camphene compound was detected in all *Salvia* samples, and the highest amounts were observed in *S. officinalis*. Eucalyptol (1,8-cineole) was detected in all *Salvia* samples, and the highest amounts of eucalyptol were observed in *S. triloba* seeds. Eucalyptol (1,8-cineole) may be considered to be one of the main components of the *Salvia* species. α-humulene was observed in all *Salvia* species except for *S. microstegia*. In total, twenty-eight compounds were identified by comparison to the mass spectra libraries NIST27 and WILEY7. Most of the determined compounds were in relatively small quantities (percentage). For instance, α-thujone, *trans*-caryophyllene, γ-terpinene, *trans*-pinocarveol, *trans*-sabinen hydrate, α-fenchyl acetate and bornyl acetate were determined to be in low amounts (%). In previous studies, β-phellandrene, thujenone, γ-terpinene and caryophyllene were reported to be rare among different *Salvia* species [8]. The results of our study were quite compatible with the literature. The headspace content of *S. nemorosa* was the richest in volatile compounds. By interpretation of the GC-MS peak area, it is possible to argue that the volatile compound content (area %) of the *Salvia* species should be arranged in the following order: *S. nemorosa > S. officinalis > S. microstegia > S. triloba > S. sclarea ≥ S. virgate.*

### 3.2. HCA and PCA of GC-MS data

**Figure 2:**
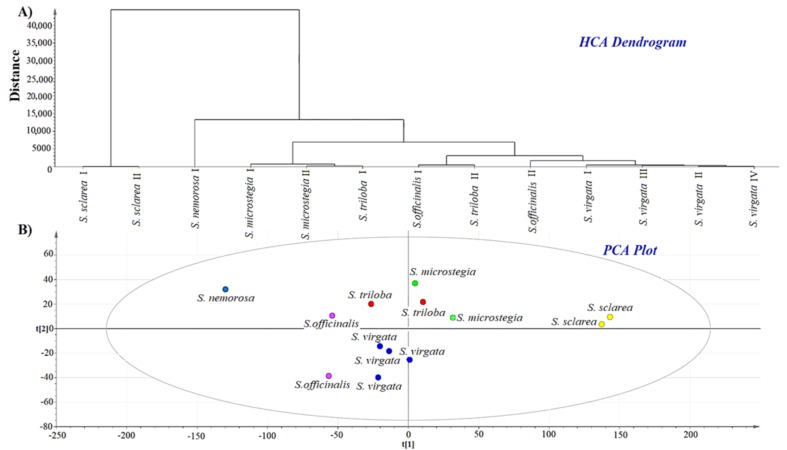
Hierarchical cluster analysis (HCA) of volatile compounds of the seed samples of *Salvia* species (**A**). Principal component analysis (PCA) of volatile compounds of the seed samples of *Salvia* species (**B**).

Principal component analysis (PCA) was performed for a total of thirteen samples of *Salvia* species using the soft independent modeling of class analogy (SIMCA). The total ion chromatograms which contained the retention times and abundances of the volatile compounds from the *Salvia* seed samples were used for the cluster analysis. *Salvia* seeds were distinguished in relation to their volatile compound compositions since the total ion chromatogram contained the fingerprint properties of the *Salvia* seed species. As seen in Figure 2B, some *Salvia* seed species were clustered with shorter distances and closer to each other. In the PCA chemometric model, two principal components explained 95% of the variance in the evaluated data. As seen in the PCA plot, *S. nemorosa* and *S. sclarea* in particular were significantly differentiated. Additionally, *S. microstegia* and *S. triloba* were clustered close to each other with relatively lower distinction. A chemometric analysis was performed using all total ion chromatograms of the *Salvia* species. According to the PCA plot, *S. sclarea* was significantly distinguished from the other species. In accordance with this, the GC-MS results showed that *S. sclarea* had the lowest volatile compound content (area %) among all species. Principal component analysis is known as an effective tool to build interrelationships between different observations such as similarities and disparities [20]. 

Additionally, an HCA analysis was performed for evaluation of the volatile fingerprints of different *Salvia* species and to evaluate the chromatographic differences of the volatile compound contents from the headspace. Chromatographic fingerprinting has been widely used and accepted by important commissions throughout the world [8]. A chromatographic fingerprint of a herb has specific information that may present the chemical characteristics of the herb [8]. The combination of the GC-MS technique with chemometrics may be effectively used for fingerprinting of various plants [21]. Ward’s algorithm was employed in the SIMCA HCA analysis. Hierarchical cluster analysis (HCA) is known as a chemometric tool that provides an opportunity to observe hidden relationships between different variables [22]. Clusters and sub-clusters are visualized precisely in dendrogram plots. As seen in Figure 2A, two well-separated groups were observed. In accordance with the PCA results, *S. sclarea* was significantly distinguished from the other *Salvia* species. The first and second samples of the same species generally clustered close to each other. *S. triloba* and *S. officinalis* samples could not be discriminated from each other in the HCA analysis. One may conclude that HCA analysis provided a deeper insight into cluster analysis since it clearly illustrated the diversity or contiguity between clusters and each integral part in a dendrogram [22]. 

The findings showed that quite compatible results were obtained using two different (HCA and PCA) analysis methods. The same classification pattern was observed in both chemometric tools. 

These findings highlighted the potential usefulness of the combination of chemometrics and GC-MS chromatographic data for distinguishing *Salvia* species on the basis of their volatile compound fingerprints. Additionally, the findings provided valuable evidence for differentiation of *Salvia* seed species.

### 3.3. FTIR-ATR Analysis

#### Characterization of FTIR spectra of *Salvia* Seeds

**Figure 3:**
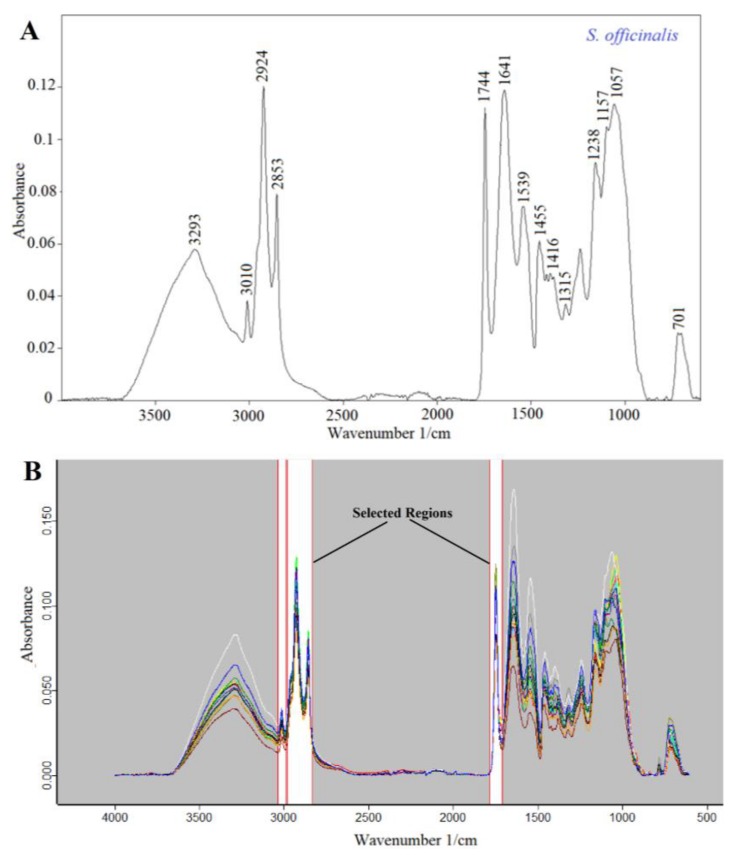
(**A**) Fingerprint Fourier transform infrared spectroscopy-attenuated total reflectance (FTIR-ATR) spectrum of *S. officinalis* seed (**B**) Overlapped FTIR spectra of all *Salvia* species.

Previous studies have reported that *Salvia* seeds are rich in fats, proteins, dietary fibers, minerals and phenolic compounds. For instance, chia seeds have 42.1 g of carbohydrates, 30.7 g of fats and 16.5 g of proteins per 100 g [6,10]. The FTIR-ATR technique may be effectively utilized for the determination of unique fingerprint characteristics of materials. Namely, it is possible to interpret the chemical composition of foods by using Fourier transform infrared spectroscopy [15]. The FTIR-ATR spectrum of *S. officinalis* seeds is presented in Figure 3A as an example. Significant bands were observed at 3293 cm^−1^, 3010 cm^−1^, 2924 cm^−1^, 2853 cm^−1^, 1744 cm^−1^, 1641 cm^−1^, 1539 cm^−1^, 1455 cm^−1^, 1416 cm^−1^, 1315 cm^−1^, 1238 cm^−1^, 1157 cm^−1^, 1057 cm^−1^ and 701 cm^−1^. These spectral bands reflect the chemical composition of *S. officinalis* seeds. In general, the bands between 3000 cm^−1^ and 3600 cm^−1^ are mainly due to the OH stretching vibrations of water [19]. The band around 3010 cm^−1^ could arise from the stretching vibrations of =C–H double bonds [20]. Two fat-related bands were observed at high intensity at 2924 cm^−1^ and 2853 cm^−1^ because of the methylene (–CH_2_–) and methyl (–CH_3_) groups, respectively [21]. Additionally, the carbonyl absorption of the triglyceride ester linkage was observed at 1744 cm^−1^ [22]. In general, the Amid I and Amid II bands result from protein-related structures. The bands observed at 1641 cm^−1^ and 1539 cm^−1^ correspond to the Amid I and Amid II bands, respectively. The spectral region between 1500 cm^−1^ and 1200 cm^−1^ includes mixed vibrations arising from the bending modes of the >CH_2_ and –CH_3_ groups in proteins, fatty acids and phosphate-bearing compounds [23]. The bands from 1220 cm^−1^ to 1011 cm^−1^ were due to the vibrations of the –C–O–C glycoside ring bond, C–O stretching in COOH and O–H bending which reveals the presence of carbohydrates and polysaccharides [24] Lastly, the spectral range between 900 cm^−1^ and 600 cm^−1^ corresponds to the true fingerprint region, and this region contains very specific spectral properties arising from aromatic amino acids and nucleotides [23].

### 3.4. Discrimination and Clustering of Salvia Seed Species Using FTIR Spectra

**Figure 4:**
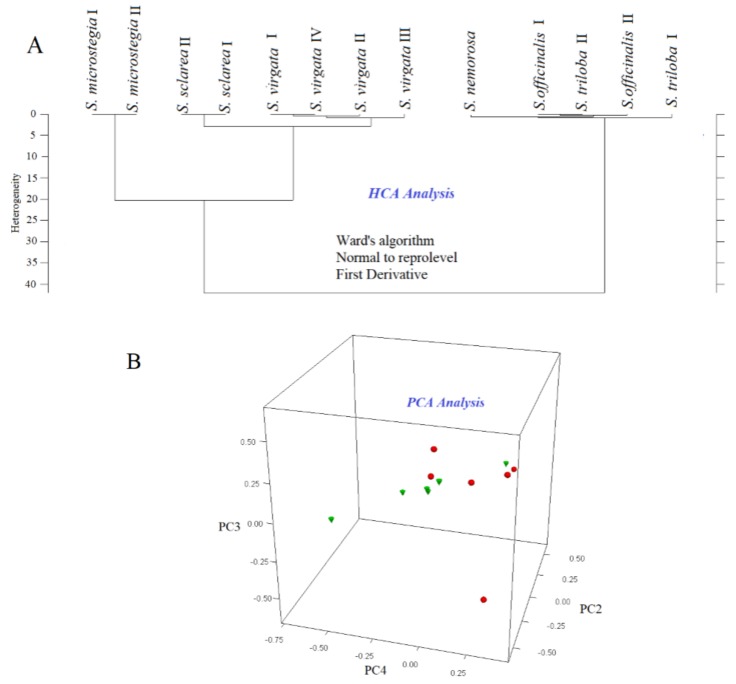
(**A**) Dendrogram of HCA (Ward’s Algorithm) of the seed samples of *Salvia* species (**B**) Three dimensional (3D) PCA map of the seed samples of *Salvia* species.

FTIR spectroscopy combined with multivariate methods such as HCA and PCA are used for arranging inspected elements into groups based on their similarities [22]. HCA and PCA analyses were performed with the aim of revealing the hidden relationships (similarities and dissimilarities) between different *Salvia* species. The FTIR spectrum of a food or any material contains very specific and characteristic information about the sample. An IR spectrum reflects the global chemical composition of the sample and can be used in the identification, characterization and quantification of the samples [23]. When we process the FTIR data using chemometrics, it becomes possible to observe hidden relationships between different samples. It is possible to build a hierarchical relationship between the samples by using HCA through algorithms. Additionally, PCA analysis was successfully performed to obtain three-dimensional (3D) score plots which presented the spectral diversity of the *Salvia* species visually. Classification of *Salvia* species was accomplished by utilizing the spectral diversity among samples through multivariate chemometric techniques. In this operation, the averaged FTIR spectra of all samples were used for chemometrics. A representative FTIR spectrum of *Salvia* species is presented in Figure 3A. In the chemometric analysis, fat-related regions (3036–2986 cm^−1^, 1782–1707 cm^−1^ and 2980–2831 cm^−1^) in the FTIR spectra were used to obtain a classification of the *Salvia* species. Additionally, selected regions are presented on overlapped spectra of all samples in Figure 3B. Classification and discrimination of all samples were accomplished using the software OPUS version 7.2 (Bruker, Germany). The chemometrics of HCA and PCA analysis is used for the arrangement of spectral data into inherent groups based on their resemblances [18]. Hierarchical cluster analysis (HCA) is an algorithmic approach that aims to construct a hierarchy of clusters. In HCA, clusters and sub-clusters are visualized in dendrogram graphs. Known as the minimum variance method, the Ward’s method joins at each stage of the cluster pair whose merger minimizes the increase in the total within-group error sum of squares, based on the normal to reprolevel spectral distance [22]. Normal to reprolevel algorithms separately calculate the spectral distances for each frequency range. The results of the HCA classification analysis of the evaluated *Salvia* species are presented in Figure 4A. As seen in the figure, two well-separated clusters were observed on the HCA dendrogram. While *S. microstegia*, *S. sclarea* and *S. virgata* clustered on the left side of the dendrogram, *S. nemorosa*, *S. officinalis* and *S. triloba* scattered on the right side of the dendrogram. As seen on the dendrogram, a high heterogeneity value was obtained (40). As a result, the HCA dendrogram revealed that the species on the same side of the dendrogram had similar fat compositions, since classification was performed on the basis of fat-related spectral bands.

Furthermore, in the principal component analysis (PCA), 3D plots of the chemometric analysis for the *Salvia* seed samples were obtained and are presented in Figure 4B. PCA was employed to display the classification (distribution) of samples as two or three-dimensional graphs. The first derivatized and vector normalized (nine smoothing points) versions of all spectra were included in classification model and a factorization algorithm was employed for calculating the spectral distances in the PCA. As shown, differently shaped and colored symbols were assigned by the chemometric model for each different species on the basis of the spectral disparities. In other words, samples were not defined as distinct groups in the software; all of the samples were loaded, and classifications and assignments were performed by the software on the basis of the selected algorithms and parameters. The same classification pattern was observed in the PCA analysis. As seen in Figure 4B, two well-separated clusters (red circles and green triangles) were observed on the PCA dendrogram. The results that were obtained showed that the two different chemometric techniques perfectly confirmed each other since the same discrimination and classification patterns were obtained in the multivariate techniques that were applied.

## 4. Conclusions 

Our study investigated the fingerprint properties of the most popular *Salvia* seed species which are grown in Cumra/Konya (Turkey) by using GC-MS and FTIR techniques. Characteristic volatile compound compositions of the *Salvia* species were obtained. Additionally, the characteristic FTIR spectra of the *Salvia* species were gained. HCA and PCA of chemometrics were performed using the GC-MS and FTIR data to reveal the hidden relationships between the *Salvia* species. In this study, for the first time, chemometrics was successfully applied to the GC-MS and FTIR data for classification of the samples in relation to their similarities and differences. In processing the FTIR data, fat-related spectral regions (3036–2986 cm^−1^, 1782–1707 cm^−1^ and 2980–2831 cm^−1^) were used to generate the dendrogram through Euclidian distance and Ward’s algorithm. In processing the GC-MS data, all total ion chromatograms were used for chemometrics. As a result, the volatile compound compositions of the *Salvia* species were determined with success. Additionally, classification and discrimination of all samples was accomplished by using multivariate techniques.

## Figures and Tables

**Table 1:** The percentage composition (area %) of identified volatile compounds in investigated the seed samples of *Salvia* species.

RI	Compounds (Area %)	*S. microstegia* I	*S. microstegia* II	*S. nemorosa* I	*S. officinalis* I	*S. officinalis* II	*S. sclarea* I	*S. sclarea* II	*S. triloba* I	*S. triloba* II	*S. virgata* I	*S. virgata* II	*S. virgata* III	*S. virgata* IV
861	*n*-hexanal	-	-	3.72 ± 0.14	1.58 ± 0.12	1.97 ± 0.51	-	-	-	-	1.51 ± 0.75	16.76 ± 1.31	19.44 ± 1.38	25.47 ± 1.65
876	*n*-hexanol	0.68 ± 0.22	-	-	0.6 ± 0.24	0.29 ± 0.62	1.1 ± 0.34	3.79 ± 0.62	0.24 ± 0.18	0.3 ± 0.12	1.72 ± 0.42	3.67 ± 0.15	2.81 ± 0.47	0.87 ± 0.72
926	tricyclene	0.18 ± 0.16	0.33 ± 0.11	-	0.72 ± 0.31	0.78 ± 0.34	-	-	0.56 ± 0.35	0.6 ± 0.67	-	-	-	-
928	α-thujene	2.24 ± 0.73	2.67 ± 0.81	32.65 ± 1.15	0.08 ± 0.05	-	-	-	-	-	-	-	-	-
939	α-pinene	36.46 ± 1.36	36.06 ± 1.52	0.63 ± 0.17	27.16 ± 1.34	25.42 ± 1.52	8.8 ± 0.91	3.81 ± 0.34	22.94 ± 0.35	24.1 ± 1.44	31.29 ± 1.68	17.8 ± 1.13	14.68 ± 1.24	17.16 ± 1.32
952	camphene	3.17 ± 0.28	3.95 ± 0.42	15.20 ± 1.82	17.81 ± 1.17	18.02 ± 1.67	3.24 ± 0.41	3.25 ± 0.67	11.52 ± 0.71	13 ± 1.74	10.9 ± 1.12	6.42 ± 0.24	4.43 ± 0.15	8.2 ± 0.14
957	2,4(10)-thujadien	0.48 ± 0.14	0.79 ± 0.13	-	-	-	-	-	-	-	-	-	-	-
978	sabinene	4.49 ± 0.47	-	2.76 ± 0.14	-	-	-	-	-	-	-	-	8.88 ± 0.71	-
979	β-pinene	16.32 ± 1.32	20.8 ± 1.5	3.05 ± 0.18	6 ± 0.82	7.59 ± 0.52	20.79 ± 1.62	21.62 ± 1.42	4.39 ± 0.51	5 ± 0.65	12.01 ± 0.43	1.66 ± 0.62	5.38 ± 0.25	9.88 ± 0.81
991	β-myrcene	0.6 ± 0.12	-	-	1.02 ± 0.17	0.71 ± 0.32	-	-	0.11 ± 0.16	0.8 ± 0.55	3.24 ± 0.27	-	-	5.34 ± 0.76
1026	α-terpinene	-	-	-	0.08 ± 0.02	0.1 ± 0.42	-	-	-	-	-	-	-	-
1029	limonen	3.09 ± 0.41	3.82 ± 0.53	28.46 ± 1.13	2.09 ± 0.25	1.94 ± 0.51	0.69 ± 0.61	1.56 ± 0.71	1.7 ± 0.51	1.2 ± 0.22	-	4.09 ± 0.58	8.55 ± 0.94	1.08 ± 0.75
1033	Eucalyptol (1,8-cineole )	24.73 ± 1.23	25.17 ± 1.12	25.03 ± 1.11	30.4 ± 1.34	32.26 ± 1.65	23.55 ± 1.72	4.17 ± 0.48	46.02 ± 0.28	45.4 ± 1.20	3.61 ± 0.63	13.99 ± 1.52	3.27 ± 0.28	2.89 ± 0.53
1060	γ-terpinene	-	0.18 ± 0.51	-	-	-	-	-	-	-	-	-		0.51 ± 0.15
1068	*trans*-sabinene hydrate	0.29 ± 0.22	0.33 ± 0.62	-	-	-	-	-	-	-	-	-	1.03 ± 0.64	0.51 ± 0.71
1100	linalool	0.26 ± 0.14	0.09 ± 0.03	-	0.19 ± 0.14	0.11 ± 0.12	3.095 ± 0.45	10.32 ± 0.56	0.08 ± 0.02	0.05 ± 0.08	-	1.31 ± 0.56	1.03 ± 0.47	-
1133	α-thujone	-	-	-	4.02 ± 0.71	-	-	-	0.1 ± 0.23	0.2 ± 0.31	3.75 ± 0.85	-	-	-
1139	*trans*-pinocarveol	0.36 ± 0.13	0.35 ± 0.26	-	5.1 ± 0.86	4.12 ± 0.28	-	-	0.28 ± 0.15	0.4 ± 0.25	1.36 ± 0.68	1.58 ± 0.47	-	0.95 ± 0.56
1143	camphor	0.45 ± 0.28		-	5.09 ± 0.72	5.59 ± 0.66	-	1.6 ± 0.19	5.86 ± 0.35	4.58 ± 1.45	2.94 ± 0.58	1.5 ± 0.34	-	0.97 ± 0.62
1165	borneol	1.42 ± 0.62	0.24 ± 0.18	-	4.17 ± 0.56	2.81 ± 0.18	2.13 ± 0.81	-	0.47 ± 0.12	0.6 ± 0.47	1.47 ± 0.61	4 ± 0.91	3.22 ± 0.87	3.43 ± 0.48
1257	linalyl acetate	0.62 ± 0.33	0.34 ± 0.33	0.3 ± 0.52	0.3 ± 0.18	-	10.0 ± 0.90	26.4 ± 0.82	0.1 ± 0.14	0.5 ± 0.21	-	-	0.8 ± 0.11	2.5 ± 0.63
1275	α-fenchyl acetate	0.71 ± 0.16	0.39 ± 0.41	2.32 ± 0.84	-	-	-	-	0.05 ± 0.01	0.09 ± 0.04	-	1.39 ± 0.25	2.53 ± 0.42	0.63 ± 0.33
1285	bornyl acetate	0.39 ± 0.32	-	0.2 ± 0.13	0.5 ± 0.43	0.26 ± 0.31	0.85 ± 0.15	7.75 ± 0.55	0.08 ± 0.02	0.06 ± 0.03	3.16 ± 0.17	2.87 ± 0.61	5.12 ± 0.71	2.97 ± 0.82
1420	*trans*-caryophyllene	-	-	0.19 ± 0.61	0.31 ± 0.87	0.33 ± 0.42	-	-	-	-	-	-	-	-
1465	α-humulene	-	-	0.12 ± 0.27	0.17 ± 0.36	0.19 ± 0.23	0.65 ± 0.37	2.63 ± 0.42	0.05 ± 0.01	0.04 ± 0.03	0.9 ± 0.05	1.13 ± 0.15	1.79 ± 0.64	0.66 ± 0.28

RI = retention index relative to *n*-alkanes (C_8_–C_40_) on Rtx-5MS fused silica capillary column (30 m × 0.25 mm (internal diameter) 0.25 μm), (-) not found.
